# Knowledge and Expectations of Perinatal Care Among Pregnant Women During the COVID-19 Pandemic

**DOI:** 10.3389/fgwh.2022.813731

**Published:** 2022-07-14

**Authors:** Caleb Chun Wei Lim, Marlene Samantha Sze Minn Goh, Ka-Hee Chua, Meei Jiun Seet, Siew Guek Tay, Manisha Mathur, Juin Yee Kong, Kee Thai Yeo

**Affiliations:** ^1^Department of Neonatology, KK Women's and Children's Hospital, Singapore, Singapore; ^2^Department of Obstetrics and Gynecology, KK Women's and Children's Hospital, Singapore, Singapore; ^3^Duke-NUS Medical School, Singapore, Singapore; ^4^Division of Nursing, KK Women's and Children's Hospital, Singapore, Singapore

**Keywords:** COVID-19, pregnancy, neonatal, breastfeeding, attitudes, perceptions, expectations, perinatal care

## Abstract

**Introduction:**

This study aimed to investigate the knowledge and expectations of pregnant women on perinatal care during the coronavirus disease 2019 (COVID-19) pandemic.

**Methods:**

A cross-sectional survey was conducted among pregnant women ≥21 years, without a history of confirmed COVID-19, attending antenatal clinics between August and September 2020 *via* a secure online platform. The survey consisted of 10 questions which evaluated the knowledge and expectations on perinatal and neonatal care during the current pandemic.

**Results:**

A total of 313 pregnant women completed the survey. The mean age of the participants was 30 years (SD 4; range 22–43 years). The median gestational age was 25 weeks (range 4–40 weeks). The participants were predominantly multiparous (54%) and almost all (98%) had completed secondary level education. Majority of participants were aware of the spread of COVID-19 by respiratory secretions and contact (90%), and the importance of prevention strategies (94%). Up to 72% agreed or strongly agreed that in-utero transmission of SARS-CoV-2 was possible. Most were unsure of the optimal mode of delivery (77%) and only 22% believed that breastfeeding was safe in a pregnant woman with active COVID-19. Although 46% were concerned about increased transmission risk with antenatal clinic visits, only 37% were agreeable to teleconferencing of clinic appointments. Maternal age >35 years was significantly associated with agreement with separation of mother-infant after birth [AOR 1.89 (95% CI 1.05, 3.39)], restrictions of visitors during the postnatal period [1.92 (1.05, 3.49)] and having their confinement practices were affected [2.3 (1.26, 4.17)]. Pregnant women who were multiparous disagreed that breastfeeding was safe in women with active COVID-19 [0.42 (0.23, 0.75)].

**Conclusions:**

There was significant uncertainty about the optimal delivery method and safety of breastfeeding with COVID-19 among expectant mothers, along with variable agreement with alterations to routine perinatal care.

## Introduction

The rapidly evolving COVID-19 pandemic resulted in much uncertainties surrounding maternal and perinatal care, leading to variations in guidelines on the management of SARS-CoV-2 infection during pregnancy and post-delivery. In the initial stages of the pandemic, most recommendations were based on expert consensus, and were of variable, low methodological rigor (1–3). As the pandemic continues to evolve, there are still remaining uncertainty about the risk and impact of COVID-19 on perinatal care (4, 5).

Due to the initial lack of consensus among many national, institutional and societal guidelines on perinatal management of pregnant women infected with SAR-CoV-2, these women faced many uncertainties in their plans for pregnancy, childbirth as well as care of their newborn. Many studies have since highlighted the psychological impact of COVID-19 precautions and alterations of care plans on pregnant women during this period (6–8). In this study, we explored pregnant women's basic understanding and knowledge of the COVID-19 infection and its impact on pregnancy. We also aimed to understand if their knowledge and perceptions affect their choices and expectations during the pre and post-delivery care, which include their openness for tele-consultations, their preferred mode of delivery, breastfeeding practices, perceived risk of social interactions with their baby and decisions on longstanding cultural birth practices in the midst of the COVID-19 pandemic.

## Subject and Methods

### Study Design and Setting

This is a single-center cross-sectional survey conducted among pregnant women attending the Specialist Obstetrics Outpatient Clinics at KK Women's and Children's Hospital (KKH), Singapore, from Aug 1, 2020 to Sept 31, 2020. KKH is Singapore's largest Obstetrics referral hospital that cares for >11,500 pregnant women annually (both inpatients and outpatients). Pregnant women aged ≥21 years old were eligible for the study – 21 years is the minimum age to provide independent consent in Singapore. Eligible women must have no documented diagnosis of COVID-19 by antigen rapid test (ART) or PCR tests, according to local guidelines for assessment of symptomatic illness or contact history if asymptomatic. Eligible and willing participants were approached by clinic staff in the patient waiting room before their antenatal appointments, to fill in a survey which was hosted on a secure online platform (FormSG, GovTech, Singapore). A quick-response (QR) code printed on a flier is scanned by the eligible women with their mobile phones in order to access the survey website and a questionnaire. All responses were obtained anonymously and participants had to provide answers for all the questions to complete the survey. The women were allowed to complete the questionnaire at their own convenience and time. The results of the survey were not revealed to the participants as the survey was conducted anonymously and participants were not tracked.

### Online Survey Tool

The online survey consisted of a set of 10 questions which were formulated based on a literature review of published international and local perinatal COVID-19 guidelines (4, 9–13) and in consultation with local obstetricians and neonatologists. The questions assessed pregnant women's knowledge regarding the modes of transmission and methods of prevention of COVID-19, as well as elicited their opinions and expectation regarding the possibility of in utero infection, optimal mode of delivery, safety of breastfeeding and teleconferencing of routine clinic visits during the current COVID-19 pandemic (Supplemental Table 1). The survey included demographic and pregnancy-related data from the participants. The majority of the survey utilized a Likert Scale (e.g. “Strongly Disagree, Disagree, Neutral, Agree and Strongly Agree”) to measure the participants' attitudes and opinions to the statements.

### Statistical Analysis

Demographic and responses to the survey were described and compared across the cohort. Multivariate analysis was performed to assess the association between demographic characteristics and opinions and the various aspects of perinatal care investigated. Adjusted odds ratios (AOR) were expressed with 95% confidence intervals (CIs). Statistical significance was set at *p* < 0.05, using a two-tailed comparison. Data was analyzed using SPSS Statistics software, version 23.0 (IBM, Armonk, New York).

### Ethics Approval

Ethics approval for this study was obtained from the SingHealth Centralized Institutional Review Board (Ref No. 2020/2648).

## Results

### Study Cohort

A total of 313 pregnant women completed the survey during the study period, representing an approximate response rate of 31% among 500 pregnant patients seen each month in the Specialist Obstetrics Outpatient Clinics. The mean age of women who participated was 30 years (SD 4), with a range from 22 to 43 years. The racial distribution of our study cohort is as follows: Chinese (54%), Malay (32%), Indian (7%; Table 1). The median gestational age of the pregnant women at survey participation was 25 weeks (range 4–40 weeks). The participants were mostly multiparous (54%) and almost all (98%) had completed secondary-level/high-school education. Around 49% of the cohort reported an annual household income >30,000 SGD (22150 USD), with 17% unemployed at the time of the survey.

**Table 1:** Clinical characteristics of survey participants.

**Demographics**	**Total (%) *n* = 313**
**Age group, years**, ***n*** **(%)**	
21–34	245 (78.3)
35 and above	68 (21.7)
**Parity**, ***n*** **(%)**	
0	146 (46.6)
1	98 (31.3)
2	49 (15.7)
3	16 (5.1)
4	4 (1.3)
**Race**, ***n*** **(%)**	
Chinese	167 (53.4)
Malay	105 (33.5)
Indian	21 (6.7)
Others	20 (6.4)
**Trimester**, ***n*** **(%)**	
1st	40 (12.8)
2nd	132 (42.1)
3rd	141 (45.0)
**Education level**, ***n*** **(%)**	
No formal education/Primary level	6 (2.0)
Secondary/high-school level	46 (14.7)
Post-Secondary level	123 (39.3)
Undergraduate	78 (24.9)
Postgraduate	60 (19.1)
**Average household income**, ***n*** **(%)**	
Unemployed	55 (17.6)
≤ SGD 10,000	42 (13.4)
SGD 10,001–30,000	63 (20.1)
SGD 30,001–50,000	70 (22.4)
SGD 50,001–80,000	35 (11.2)
≥SGD 80,001	48 (15.3)

### Participants Responses to Survey Questions

The breakdown of responses to questions regarding knowledge, understanding and expectations of perinatal and neonatal care is summarized in Tables 2, 3 respectively.

1. Knowledge and Understanding of Perinatal and Neonatal Care

a) Transmission & Prevention – A large proportion of survey participants (90%) agreed that COVID-19 is spread predominantly by airway secretions and by contact with these secretions. Up to 94% also agreed that wearing a mask and frequent hand hygiene could prevent the spread of COVID-19.
b) Vertical transmission – Majority of pregnant women surveyed (72%) agreed or strongly agreed that the transmission of COVID-19 to the unborn fetus during pregnancy was possible. The proportion of women with this belief about possible in utero transmission was consistent regardless of parity (68.9% multiparous and 76% of nulliparous women agreed), gestation at time of survey (70.0% of women in the first trimester, 73.7% of women in the second trimester, and 70.9% of women in the third trimester agreed), age (72.2% of women <34 years and 72.1% of women >35 years agreed), income levels (72.2% with earnings < SGD 10,000 and 72.3% with >SGD 50,001 agreed) or education level (73.1% educated up to secondary level and 68.3% of women with a post-secondary degree agreed).
c) Mode of delivery – The majority of survey participants (77%) were unsure about the optimal and safe delivery method for pregnant women with active COVID-19. Up to 17% of the participants believed that normal vaginal delivery was a safe option. The proportion of women with uncertainty regarding safe delivery method was reasonably consistent when stratified by parity (70.7% of multigravida women and 83.6% of primigravida women were unsure), gestation (77.5% of women in their first trimester, 77.6% of women in their second trimester and 75.2% of women in their third trimester were unsure), age (70.6% of women <34 years and 78.4% of women >35 years were unsure), income levels (81.4% with earnings < SGD 10,000 and 66.3% with >SGD 50,001 were unsure) or education level (76.9% of women who were educated up to secondary level and 78.9% of women who obtained a post-secondary degree were unsure).
d) Breastfeeding with COVID-19 – Slightly more than half (53%) of the participants were unsure if it were safe for a mother with COVID-19 to breastfeed. Another 24.6% of pregnant women thought that breastfeeding should be avoided altogether. Subgroup analysis revealed that this uncertainty surrounding breastfeeding was consistent across participants regardless of whether they were stratified by parity (58.9% of multigravida women and 47.9% of primigravida women were unsure), gestation (47.5% of women in the first trimester, 53.8% of women in the second trimester and 53.8% of women in the third trimester were unsure), age (51% of women <35 years and 60.3% of women >35 years were unsure), income levels (54.6% with earnings < SGD 10,000 and 57.8 % with earnings >SGD 50,001 were unsure) or education levels (57.7% of women who were educated up to secondary level and 56.9% of women who obtained a post-secondary degree were unsure).

2. Expectations of Perinatal & Neonatal Care

a) Concerns about hospital visits and risk of COVID-19 – The majority of respondents (46.6%) were concerned about the risk of contracting COVID-19 at the hospital when appearing for their routine appointments or during labor. When asked about teleconferencing routine clinic appointments, 37% of study participants were open to the idea, whereas 28% of the study cohort disagreed. Higher proportion of women who were >35 years of age (29.4% vs. 27.8%, *p* = 0.07) and those who were primigravida (31.5% vs. 25.1%, *p* = 0.1) rejected teleconferencing. Patients in their first trimester were more likely to be open to the idea of teleconferencing (55%) compared to those in their second (35.3%) and third trimesters (34.2%), *p* = 0.06. Around 59% of survey participants also agreed or strongly agreed that restriction of visitors during the period of labor was necessary amid the COVID-19 pandemic. Even so, 18% of the participants strongly disagreed with the restriction of visitors.
b) Postnatal and Confinement practices - More than half of the pregnant women surveyed (56.5%) agreed or strongly agreed that prevailing confinement practices would be disrupted by the COVID-19 pandemic, while 13.7% of the cohort did not perceive that their confinement practices would be affected. This expectation did not differ with parity, with 55.1% of primiparous participants and 58.2% of multiparous participants reporting that their confinement practices will be affected.

**Table 2:** Knowledge and understanding of perinatal and neonatal care during the current COVID-19 pandemic.

**Questions**	**Responses**	**Total, *n* (%)**
Q1. COVID-19 is mainly spread	Strongly agree/agree	283 (90.4)
by airway secretions from infected	Neutral	26 (8.3)
persons and by contact with these secretions	Strongly disagree/disagree	4 (1.3)
Q2. Wearing a mask and frequent hand hygiene can help to prevent the spread of COVID-19	Strongly agree/agree Neutral Strongly disagree/disagree	294 (93.9) 15 (4.8) 4 (1.3)
Q3. There is a risk of spreading	Strongly agree/agree	226 (72.2)
COVID-19 to the unborn child if a	Neutral	61 (19.5)
pregnant woman is infected with COVID	Strongly disagree/disagree	26 (8.3)
Q4. It is safe for women with	Normal vaginal delivery	53 (16.9)
COVID-19 to deliver their baby	Cesarean section	20 (6.4)
*via*	Unsure	240 (76.7)
Q5. Breastfeeding in mothers with COVID-19 is	Safe and should be encouraged	70 (22.4)
	Risky and should be avoided	77 (24.6)
	Unsure	166 (53.0)
Q6. For mothers with COVID-19	Strongly agree/agree	169 (54.0)
separation of the mother and	Neutral	94 (30.0)
infant after birth and for up to 14 days is necessary to prevent infection of the infant	Strongly disagree/disagree	50 (16.0)

**Table 3:** Expectations of perinatal and neonatal care during the current COVID-19 pandemic.

**Questions**	**Responses**	**Total, *n* (%)**
**Q7**. I am worried about the risk	Strongly agree/agree	146 (46.6)
of contracting COVID-19 when I	Neutral	108 (34.5)
come to hospital for my routine check-ups and delivery	Strongly disagree/disagree	59 (18.9)
**Q8**. I would be open to the idea of	Strongly agree/agree	117 (37.4)
teleconferencing my clinic	Neutral	108 (34.5)
appointments to minimize the physical visits to the hospital	Strongly disagree/disagree	88 (28.1)
**Q9**. I would accept restriction of	Strongly agree/agree	185 (59.1)
visitors during the labor, delivery	Neutral	72 (23.0)
and postnatal stay due to the COVID 19 situation	Strongly disagree/disagree	56 (17.9)
**Q10**. My plans for confinement	Strongly agree/agree	177 (56.5)
practices will be affected/have been	Neutral	93 (29.7)
affected by restrictions and hygiene recommendations during the current COVID-19 situation	Strongly disagree/disagree	43 (13.7)

### Factors Affecting Knowledge and Expectation of Perinatal and Neonatal Care

The associations between sociodemographic factors and maternal understanding and expectations of perinatal and neonatal care during the current pandemic are shown in Table 4. There was no significant association between the sociodemographic factors evaluated and maternal agreement with the possibility of in-utero transmission of COVID-19 and the risk of vaginal delivery in mothers with COVID-19. Maternal age >35 years was significantly associated with agreement with separation of mother-infant after birth [AOR 1.89 (95% CI 1.05, 3.39)], restriction of visitors during postnatal period [1.92 (1.05, 3.49)] and that their confinement practices were affected [2.3 (1.26, 4.17)]. Pregnant women who were multigravidas disagreed or strongly disagreed that breastfeeding was safe in women with active COVID-19 [0.42 (0.23, 0.75)].

**Table 4:** Multivariable analysis of factors affecting pregnant women's understanding and expectations of perinatal and neonatal care.

	**There is a risk of spreading COVID-19 to the unborn child if a pregnant woman is infected with COVID** **AOR (95%CI)**	**For mothers with COVID-19, separation of the mother and infant after birth and for up to 14 days is necessary to prevent infection of the infant** **AOR (95%CI)**	**Breastfeeding in mothers with COVID-19 is safe and should be encouraged** **AOR (95%CI)**	**It is safe for women with COVID-19 to deliver their baby *via* normal vaginal delivery** **AOR (95%CI)**	**I would be open to the idea of teleconferencing my clinic appointments to minimize the physical visits to the hospital** **AOR (95%CI)**	**I would accept restriction of visitors during the labor, delivery and postnatal stay due to the COVID 19 situation** **AOR (95%CI)**	**My plans for confinement practices will be affected/have been affected by restrictions and hygiene recommendations** **AOR (95%CI)**
**Education level**							
Primary level and under	Ref	Ref	Ref	Ref	Ref	Ref	Ref
Post-secondary level	0.75 (0.36, 1.59)	0.91 (0.45, 1.83)	0.97 (0.42, 2.27)	0.87 (0.35, 2.17)	1.58 (0.77, 3.27)	1.57 (0.79, 3.13)	0.77 (0.39, 1.53)
Undergraduate and above	1.07 (0.47, 2.43)	0.80 (0.38, 1.69)	1.51 (0.62, 3.69)	0.90 (0.34, 2.41)	1.19 (0.55, 2.59)	2.57 (1.21, 5.45)*	1.14 (0.55, 2.37)
**Pregnancy trimester**							
1	Ref	Ref	Ref	Ref	Ref	Ref	Ref
2	1.18 (0.55, 2.57)	0.82 (0.40, 1.68)	0.44 (0.19, 1.00)	0.65 (0.26, 1.61)	0.47 (0.23, 0.96)	0.58 (0.27, 1.23)	1.57 (0.76, 3.23)
3	1.01 (0.46, 2.25)	1.89 (0.89, 4.02)	0.77(0.34, 1.74)	0.93 (0.37, 2.34)	0.45 (0.21, 0.95)	0.90 (0.41, 1.96)	1.62 (0.76, 3.41)
**Gravidity**							
Primigravida	Ref	Ref	Ref	Ref	Ref	Ref	Ref
Multigravida	1.3 (0.85, 2.42)	1.50 (0.92, 2.43)	0.42 (0.23, 0.75)*	0.56 (0.30, 1.06)	1.21 (0.75, 1.97)	0.83 (0.51, 1.34)	1.14 (0.70, 1.83)
**Age groups, years**							
21–34	Ref	Ref	Ref	Ref	Ref	Ref	Ref
35 and above	1.05 (0.57, 1.95)	1.89 (1.05, 3.39)*	0.56 (0.27, 1.15)	1.14 (0.56, 2.30)	1.0 (0.56, 1.79)	1.92 (1.05, 3.49)*	2.3 (1.26, 4.17)*
**Annual income**							
≤ SGD 10,000	Ref	Ref	Ref	Ref	Ref	Ref	Ref
SGD 10,001–50,000	1.04 (0.57, 1.89)	1.95 (1.11, 3.40)*	0.98 (0.50, 1.93)	1.03 (0.48, 2.23)	1.53 (0.87, 2.69)	0.91 (0.52, 1.58)	1.24 (0.72, 2.14)
≥SGD 50,001	0.86 (0.41, 1.79)	2.09 (1.06, 4.13)*	1.34 (0.60, 2.96)	2.32 (0.97, 5.52)	1.27 (0.6, 2.52)	0.68 (0.34, 1.34)	1.61 (0.82, 3.17)

*Results shown combines association with those who agree or strongly agree*.

*p < 0.05.

## Discussion

In this study, we explored the knowledge and expectations of perinatal and neonatal care among pregnant mothers during the current COVID-19 pandemic in Singapore. Our survey revealed that most participants were aware of the modes of SARS-CoV-2 transmission and the important transmission prevention strategies. There was significant uncertainty identified in their understanding of the safe mode of delivery and of breastfeeding in mothers with COVID-19. Up to 60% of those surveyed were neutral or disagreed with alterations to pre-pandemic standards of perinatal care including the use of teleconferencing, separation of mother and infant after birth, restriction of in-hospital visitors and alterations to confinement practices.

There are limited current studies reporting on the perception and expectations of perinatal care by pregnant women's during the current COVID-19 pandemic. In a recent study of the perception of the impact of COVID-19 on pregnancy and psychological impact on pregnant women, Ng et al. highlighted the importance of timely, accurate information on the impact of COVID-19 on pregnancy and its effect on the psychological well-being of pregnant women (14). Knowledge gaps in this regard among antenatal women were associated with increased anxiety and depression during this current pandemic. In a national cross-sectional survey conducted in Italy, Ravaldi et al. (6) reported significant changes in pregnant women's expectations regarding childbirth where they expressed more fear, anxiety, pain and loneliness during this current pandemic. They also found that women with a history of psychological distress were significantly more likely to be overwhelmed by the situations caused by the COVID-19 pandemic (6, 7). Another study on COVID-19 awareness among pregnant women revealed that social demographic factors such as maternal age, ethnicity, frontline jobs and attendance at high-risk clinics are likely to influence the attitudes and precaution practices among of pregnant women (15). All these studies highlight the importance of appropriate and targeted counseling to pregnant women on the potential effect of COVID-19 on pregnancy as a measure of psychological support. Our study adds to this by illustrating the potential importance of early and appropriate provision of evidence-based information to expectant mothers to reduce misinformation and moderate their expectations of perinatal and neonatal care during this current pandemic.

Much of the anxiety among pregnant women may be related to the variability of recommendations on perinatal and neonatal care that was available during the early phases of the pandemic. Most recommendations were based on expert consensus with limited evidence which were of variable and low methodological rigor (1–5). This was likely inevitable considering the speed and magnitude of the pandemic and the rapidly evolving nature of the evidence that was available. This is evidenced by the emerging evidence on transmission of SARS-CoV-2 in utero. Recent data have confirmed the possibility of in-utero transmission, even though this is likely a very rare occurrence (16–18). This emerging information was reflected in the majority of our survey participants agreeing that in-utero transmission of SARS-CoV-2 virus to the unborn fetus was possible. This could also possibly be due to the widespread coverage of reports of newborns diagnosed and infected with COVID-19 shortly after birth (19, 20). However, current evidence shows that the risk of vertical transmission of SARS-CoV-2 is low and a cesarean section may not prevent vertical transmission (21).

Delivery room practices are important to mitigate the potential risk of perinatal SARS-CoV-2 transmission (22–25). Emerging reports have reported SARS-CoV-2 being detected in amniotic fluid, vaginal fluid and the placenta (17, 18), highlighting the possibility of infection in utero and during delivery. Systematic review of cases reported in the literature has indicated no substantial evidence for increased transmission risk during vaginal birth (21, 26–28). Even so, most of the women in our cohort had expressed uncertainty regarding the optimal mode of delivery in women with COVID-19. While only 22% of women held a definitive opinion, 6% would choose to have a cesarean section and 16% would choose to have a vaginal delivery. This is in contrast to another earlier study (15) which showed that 53% of women would opt to have a Cesarean section over a vaginal delivery if infected with SARS-CoV-2. This may be a reflection of the evolving and emerging evidence, especially that being shared in the media and by international perinatal organizations (9, 10, 12). This uncertainty should be addressed and communicated by the clinicians, as the evidence for the safety of routine obstetric indications for delivery of pregnant women with COVID-19 accumulates (13, 29). Current evidence maintains that mode of delivery should be based on standard obstetric indications even in the face of an active COVID-19 infection as the rate of vertical or peri-partum transmission of SARS-CoV-2 is low (21, 26–28). Furthermore, nosocomial COVID-19 infection can further be avoided with strict peripartum protocols (29, 30).

Breastfeeding and the feeding of mother's own breast milk by women with COVID-19 have also been areas of significant contention with significant variability in the initial guidelines (1, 2, 30). Only 22% of our study cohort would choose to breastfeed their infant with active COVID-19, where the remaining 78% were unsure or would totally avoid breastfeeding altogether. This finding is consistent to that reported by Yassa et al. (31), where 50% of the women surveyed was unsure if breastfeeding was safe during the pandemic. Breastfeeding and provision of breast milk, with its well-documented short and long-term health benefits, is an important aspect that needs to be addressed (11, 32). While there have been a small number of studies that have reported breastfed infants testing positive for SARS-CoV-2 (17, 33, 34), the mode of infection (whether through close contact with their mothers or through the consumption of breast milk itself) cannot be conclusively determined. The lack of viable virus detected in reverse transcription polymerase chain reaction (RT-PCR) positive breast milk (35) combined with the presence of SARS-CoV-2 specific immunoglobulin A response in breast milk samples (36) after COVID-19, suggests the low likelihood of transmission. Current evidence and guidelines recommend breastfeeding as usual even in the face of a COVID-19 infection, as long as the appropriate precautionary measures (e.g. wearing a mask and performing hand hygiene) are taken.

While 46.6% of participants expressed concern about the risk of contracting COVID-19 during their hospital visits, only 37% were receptive to the idea of teleconferencing as an option. Notably, women who were nulliparous were less likely to agree. Teleconferencing confers increased autonomy to the patient but would also rely heavily on patient involvement and reporting (37). It is likely that nulliparous women are likely to have less confidence on self-monitoring and reporting of issues during her pregnancy. Pregnant women >35 years were also less likely to agree to teleconferencing of hospital visits. This may be related to the perceived increased risk associated with advanced age pregnancies. With the potential need for ongoing social distancing procedures during this pandemic and beyond (38), there needs to be increased effort to improve the knowledge and increase the comfort level of pregnant women for home monitoring through the potential implementation of suitable monitoring devices and applications (39, 40).

Confinement is a unique postnatal practice specific to several Asian ethnicities and communities which involves the prohibition of performing certain daily tasks and the restriction of certain foods and diets. Local studies have previously shown that a negative postnatal confinement experience was a significant risk factor for postnatal depression (41). More than half of the women surveyed reported that their confinement plans were being affected by restrictions imposed due to the current pandemic. In this regard, physicians must be aware of the importance of confinement especially in the Asian context and its significant contribution to the overall pregnancy experience. Being cognizant of the different confinement activities practiced by different ethnicities (42) and how these activities may be affected by the COVID-19 pandemic, is important to provide tailored advice to pregnant women on the postpartum care for themselves as well as for their neonates. This would also aid in improving the doctor-patient relationship and the outcomes for both pregnant women and their neonates.

Our study is limited by our relatively small sample size as well as the conduct of the survey in a single hospital in Singapore. Even so, our study cohort was recruited from the largest perinatal center on the country and the ethnic distribution is well representative of the general Singaporean population (43). Our findings could have been influenced by selection bias as our survey was conducted on a voluntary basis and on an online platform requiring mobile devices. This could have inevitably excluded those less technologically savvy or pregnant women without mobile devices. Other reasons for non-participation could include lack of interest and priority, focus on their clinical consultation and lack of time or unfamiliarity of answering a survey. Active real time engagement of participants by study team member in the clinic, helping them through the survey questions could have helped those who had language and/or device barrier. To expand our study further, the translation of our survey into more languages and expanding the scope to neighboring South-East Asian countries to see if the findings are reproducible could be explored.

Our study did not specifically collect information on the presence of any pregnancy complications from the participants. As such, we were unable to determine if the presence of such complications could have influenced the responses provided. In addition, the lack of a qualitative “free-text” portion to the survey and/or a focus group discussions limited the amount of data obtained. This would have allowed participants to further elaborate on their concerns and impetus behind the responses given, including complications such as malformations, miscarriage, and/or still births. We omitted direct questions regarding risks of malformations in the unborn child as a result of COVID-19 so as not to instill fear or anxiety in the women. Information on such complication during pregnancy was still relatively unclear at that point of time.

Our survey revealed significant uncertainties by expectant mothers in relation to the mode of delivery and safety of breastfeeding in pregnancies complicated by COVID-19, along with variability to the agreement with alterations to the perinatal care. This study highlights the importance of providing timely, evidence-based information to the pregnant women and the general public to increase their knowledge and modulate the expectations of perinatal care during this current pandemic. The results of this survey also highlight key areas of concern with regards to COVID-19 in pregnancy. Every effort has to be made by medical professionals to educate pregnant women on the latest COVID-19 guidelines and evidence so as to clear any misconceptions regarding important aspects of perinatal care such as breastfeeding.

## Data Availability Statement

The original contributions presented in the study are included in the article/Supplementary Material, further inquiries can be directed to the corresponding author.

## Ethics Statement

The studies involving human participants were reviewed and approved by SingHealth Centralized Institutional Review Board. Written informed consent for participation was not required for this study in accordance with the national legislation and the institutional requirements.

## Author Contributions

CL, MG, JK, and KY conceived the study, acquired the data and contributed to the analysis and drafting of the manuscript. ST, K-HC, MS, and MM contributed to the acquisition of data and critically revised the manuscript. All authors read and approved the final manuscript.

## Conflict of Interest

The authors declare that the research was conducted in the absence of any commercial or financial relationships that could be construed as a potential conflict of interest.

## Publisher's Note

All claims expressed in this article are solely those of the authors and do not necessarily represent those of their affiliated organizations, or those of the publisher, the editors and the reviewers. Any product that may be evaluated in this article, or claim that may be made by its manufacturer, is not guaranteed or endorsed by the publisher.
