# Supplementary material for: Knowledge and Expectations of Perinatal Care Among Pregnant Women During the COVID-19 Pandemic
Source: Front Glob Womens Health. 2022 Jul 14;3:813731. doi: 10.3389/fgwh.2022.813731 (PMC9329695; doi:10.3389/fgwh.2022.813731)
Supplement: Supplementary file 1 [file Table_1.DOCX]

**Supplemental Table 1. Online Survey Tool Questions**

**Demographic Information**

| What is your age?  (free response box) |
| --- |
| What is your Estimated Due Date (EDD)?  (free response box) |
| What is your Ethnicity?  (choice between Chinese, Malay, Indian, Eurasian, Others) |
| What is your highest education level?  (choice between No formal education, Primary, Secondary, Tertiary/Junior College/Polytechnic, Undergraduate, Postgraduate) |
| What is your average annual household income?  (choice between unemployed, ≤ SGD 10000, SGD 10001 – 30000, SGD 30001 – 50000, SGD 50001 – 80000 and ≥ SGD 80001) |
| How many children have you given birth to before? Please exclude this current pregnancy.  (free response box) |

**Survey Questions**

| 1. COVID-19 is mainly spread by airway secretions from infected persons and by contact with these secretions  (Likert scale : Strongly agree – Agree – Neutral – Disagree – Strongly Disagree) |
| --- |
| 2. Wearing a mask and frequent hand hygiene can help to prevent the spread of COVID-19  (Likert scale : Strongly agree – Agree – Neutral – Disagree – Strongly Disagree) |
| 3. There is a risk of spreading COVID-19 to the unborn child if a pregnant woman is infected with COVID  (Likert scale : Strongly agree – Agree – Neutral – Disagree – Stro ngly Disagree) |
| 4. It is safe for women with COVID-19 to deliver their baby   1. By normal vaginal route 2. by Caesarean section 3. Unsure |
| 5. Breastfeeding in mothers with COVID-19   1. Is safe and should be encouraged 2. is risky and should be avoided 3. Unsure |
| 6. For mothers with COVID-19, separation of the mother and infant after birth and for up to 14 days is necessary to prevent infection of the infant  (Likert scale : Strongly agree – Agree – Neutral – Disagree – Strongly Disagree) |
| 7. I am worried about the risk of contracting COVID-19 when I come to hospital for my routine check-ups and delivery  (Likert scale : Strongly agree – Agree – Neutral – Disagree – Strongly Disagree) |
| 8. I would be open to the idea of teleconferencing my clinic appointments to minimise the physical visits to the hospital  (Likert scale : Strongly agree – Agree – Neutral – Disagree – Strongly Disagree) |
| 9. Would you accept restriction of visitors during the labour, delivery and postnatal stay due to the COVID-19 situation?   1. Yes 2. No 3. Does not matter |
| 10. My plans for confinement practices will be affected/have been affected by restrictions and hygiene recommendations during the current COVID-19 situation  (Likert scale : Strongly agree – Agree – Neutral – Disagree – Strongly Disagree) |
